# Mining Public Data to Investigate the Virome of Neglected Pollinators and Other Floral Visitors

**DOI:** 10.3390/v15091850

**Published:** 2023-08-31

**Authors:** Sabrina Ferreira de Santana, Vinícius Castro Santos, Ícaro Santos Lopes, Joel Augusto Moura Porto, Irma Yuliana Mora-Ocampo, George Andrade Sodré, Carlos Priminho Pirovani, Aristóteles Góes-Neto, Luis Gustavo Carvalho Pacheco, Paula Luize Camargos Fonseca, Marco Antônio Costa, Eric Roberto Guimarães Rocha Aguiar

**Affiliations:** 1 Department of Biological Science, Center of Biotechnology and Genetics, Universidade Estadual de Santa Cruz, Ilhéus 45662-900, BA, Brazil; sfsantana@uesc.br (S.F.d.S.);; 2 Department of Biochemistry and Immunology, Universidade Federal de Minas Gerais, Belo Horizonte 31270-901, MG, Brazilarigoesneto@gmail.com (A.G.-N.); 3 Department of Microbiology, Institute of Biological Sciences, Universidade Federal de Minas Gerais, Belo Horizonte 31270-901, MG, Brazil; 4 Department of Biotechnology, Institute of Health Sciences, Universidade Federal da Bahia, Salvador 40231-300, BA, Brazil; 5 Department of Genetics, Institute of Biological Sciences, Universidade Federal de Minas Gerais, Belo Horizonte 31270-901, MG, Brazil

**Keywords:** virome, pollinators, insects, *Malvaceae*

## Abstract

This study reports the virome investigation of pollinator species and other floral visitors associated with plants from the south of Bahia: *Aphis aurantii*, *Atrichopogon* sp., *Dasyhelea* sp., *Forcipomyia taiwana*, and *Trigona ventralis hoozana*. Studying viruses in insects associated with economically important crops is vital to understand transmission dynamics and manage viral diseases that pose as threats for global food security. Using literature mining and public RNA next-generation sequencing data deposited in the NCBI SRA database, we identified potential vectors associated with *Malvaceae* plant species and characterized the microbial communities resident in these insects. Bacteria and Eukarya dominated the metagenomic analyses of all taxon groups. We also found sequences showing similarity to elements from several viral families, including *Bunyavirales, Chuviridae, Iflaviridae*, *Narnaviridae, Orthomyxoviridae, Rhabdoviridae, Totiviridae*, and *Xinmoviridae*. Phylogenetic analyses indicated the existence of at least 16 new viruses distributed among *A*. *aurantii* (3), *Atrichopogon* sp. (4), *Dasyhelea* sp. (3), and *F. taiwana* (6). No novel viruses were found for *T. ventralis hoozana*. For *F. taiwana*, the available libraries also allowed us to suggest possible vertical transmission, while for *A. aurantii* we followed the infection profile along the insect development. Our results highlight the importance of studying the virome of insect species associated with crop pollination, as they may play a crucial role in the transmission of viruses to economically important plants, such as those of the genus Theobroma, or they will reduce the pollination process. This information may be valuable in developing strategies to mitigate the spread of viruses and protect the global industry.

## 1. Introduction

Pollination is a fundamental ecological process that underpins the reproduction of angiosperms and ensures the production of fruits, seeds, and ultimately, the survival of numerous plant species [1,2]. It is a vital interaction between plants and pollinators, which can be insects, birds, bats, and even other animals, facilitating the transfer of pollen from the male reproductive organs to the female reproductive organs of flowers [3,4]. This process not only enables the successful fertilization and genetic diversity of plant populations, but also provides significant benefits to ecosystems, agriculture, and the global food supply. It is a crucial process in plant reproduction, involving the transfer of pollen to the stigma for fertilization and fruit formation. It can occur through cross-pollination, where pollen is transferred between different plants, or self-pollination, where pollen is transferred within the same flower or plant. Pollination can be achieved by biotic agents, such as animals and insects, or abiotic agents, such as wind or water [5]. In the case of insect pollination, several insect species play a vital role in ensuring successful pollination and subsequent fruit production [6,7,8,9,10,11,12].

Insect pollinators, particularly flower-visiting species, have been identified as important contributors to pollination services. These insects facilitate the transfer of pollen between flowers, thereby promoting fertilization and fruit set. Additionally, they contribute to ecosystem functioning, nutrient cycling, and maintenance of soil quality [3]. The presence of effective pollinators is especially crucial for plant species that have specific pollination requirements.

While the importance of well-known pollinators such as bees, butterflies, and birds cannot be underestimated, it is essential to recognize that there are numerous, other, less-studied pollinators that also contribute significantly to the pollination process. These include flies, beetles, wasps, and even mammals like bats, rodents, and primates [13,14]. In this context, several insects have been observed as visitors to flowers, including *Aphis* (*Toxoptera*) *aurantii*, black ants (*Dolichoderus bituberculatus*), mealybugs, Anoplolepis, drosophilid flies, Trigona bees, gall midges, Ceratopogonidae mosquitoes, and others [15]. Among these less-studied pollinators, limited research has focused on characterizing their microbiomes and viromes. For instance, *Aphis aurantii*, commonly known as the orange aphid, is a sap-sucking insect that feeds on various plant species, including citrus trees. Despite being recognized as a potential vector for transmitting plant viruses, studies elucidating its associated microbiome and virome remain scarce or restricted to the transmission of *Citrus tristeza virus* [16]. Similarly, other flower-visiting insects like *Dasyhelea* sp., *Atrichopogon* sp., *Forcipomyia taiwana*, and *Trigona ventralis hoozana*, representing distinct groups, have received little attention regarding their microbial and viral communities.

Although some of these insect species, such as Ceratopogonidae mosquitoes, have been casually observed visiting flowers in some cultures, their effectiveness as pollinators and as subsequent fruiting support are yet to be fully established [17,18,19,20]. Nonetheless, it is important to note that these less studied pollinators still play significant roles in environmental quality due to their foraging habits and interactions with plant reproduction. Insects of the genera *Atrichopogon*, *Dasyhelea*, and *Forcipomyia* have also been identified as potential pollinators based on their morphological traits [10,21,22]. For example, *Dasyhelea flava* has been found to feed on both honeydew and plant sugar solutions, occasionally visiting flowers in search of nectar [23]. Trigona bees, both larger and smaller-sized, have been recognized as floral pollinators or occasional visitors in some plants [24]. 

Beyond their role in pollination, some insects can also act as potential vectors of viruses. For example, species of the genera *Atrichopogon*, *Ceratopogonidae*, *Dasyhelea* and *Forcipomyia* have been associated with the transmission of arboviruses and parasites [25,26]. However, their role in transmitting plant viruses has not been fully understood thus far. It is worth mentioning that most studies on viruses in pollinators have primarily focused on bees, neglecting other potential pollinators and associated insects in the plant–flower microenvironment.

Understanding the microbiomes and viromes of neglected flower visitors is of utmost importance for several reasons. First, characterizing their microbial and viral compositions can provide valuable insights into the functional roles of specific microorganisms and viruses in pollination dynamics, plant health, and disease transmission. For instance, the microbiome of flower visitors may contribute to their ability to efficiently extract and digest floral resources, while the virome might influence viral transmission dynamics among plants [27]. Second, the microbial and viral communities associated with neglected insects could significantly impact their overall fitness, behavior, and adaptation to their specific ecological niches. Indeed, symbiotic bacteria present in the microbiome of insects can provide essential nutrients and confer resistance against pathogens [28,29]. Third, neglected flower visitors may serve as potential reservoirs of beneficial or pathogenic microorganisms and viruses, which can be transferred to and impact other organisms within the floral ecosystem.

Therefore, studying the diversity and composition of viruses in these insect species associated with crops is essential to better understand the transmission dynamics and management of viral diseases that can affect economically important plants [30]. In our study, we investigated the virome of these neglected flower visitors using publicly available RNA-Seq libraries, identifying several non-retroviral exogenous sequences. These findings underscore the importance of exploring the virome of insect species associated with crops, as they can potentially contribute to virus transmission and impact crop productivity.

## 2. Materials and Methods

### 2.1. Species Selection

**Figure 1:**
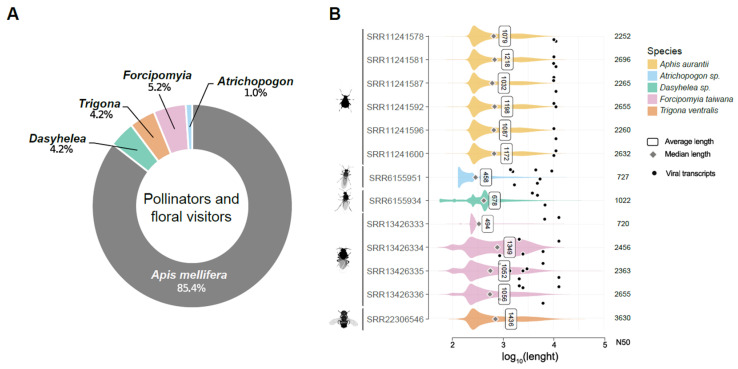
Overview of public data on plants and associated insects. (**A**) Insect species often associated with plant pollination and other plant-visiting insects referred to in the literature search. (**B**) Transcriptome assembly statistics for each library referring to insects associated with plants For each library, the value on the right column indicates N50, while the white box represents the mean and the diamond is related to the median value of the transcripts assembled. Black dots represent viral transcripts selected for further characterization.

We searched articles on PubMed (https://pubmed.ncbi.nlm.nih.gov/, accessed on 10 July 2023) using “pollinators” AND “plants” as key words occurring in title and/or abstract. The list was manually curated by screening the main text for selecting only original manuscripts investigating pollination of economically important plant species with a focus on those grown in Brazil. This inspection led to the selection of 96 articles that were included in this research (Supplementary Table S1). From the obtained data, we processed the resulting file to allow the creation of visual representations according to their geographical origin, as well as an overview of the production by date and the main insects and plants analyzed in these studies. Therefore, species of pollinators and floral visitors associated with economically important plants that lack studies on their virome were selected for investigation (Figure 1).

### 2.2. Retrieval of RNA-Seq Libraries

Public RNA-Seq libraries were downloaded from the Sequence Read Archive (SRA) using the SRA Toolkit (version 3.0.3) from the NCBI database. The libraries of *Aphis aurantii* (SRR11241578, SRR11241581, SRR11241587, SRR11241596, SRR11241592, and SRR11241600) are derived from transcriptomic sequencing of the developmental stages [31]. The libraries of *Atrichopogon* sp. (SRR6155951) and *Dasyhelea* sp. (SRR6155934) are derived from field samples collected in Australia and Singapore, respectively [23]. The libraries of *F. taiwana* (SRR13426333, SRR13426334, SRR13426335, and SRR13426336) belong to a transcriptome profiling study whose goal was to reveal the developmental regulation of *F. taiwana* eggs treated with NaCl [32]. The libraries of *Trigona ventralis hoozana* (SRR5601896 and SRR5601830) do not have associated publications (Supplementary Table S2).

### 2.3. Bioinformatic Analysis

#### 2.3.1. Pre-Processing, Integrative Assembly, and Viral Sequences Identification

The quality of each RNA-Seq library was checked using FastQC (version 0.11.9) [33] and filtered using CutAdapt (version 4.2) [34]. As the host reference genome was not available for the insects investigated, and considering the possibility that insect sequences might predominate due to the lack of complete host removal, we tentatively employed genomes from taxonomically related pollinating species as a surrogate. Specifically, we used *Culicoides tainanus* [GCA_020740445.1] and *Apis mellifera* [GCA_003254395.2] genomes as alternatives to compensate for the unavailability of reference genomes for the species under investigation. The previously selected RNA-Seq libraries were aligned to reference sequences using the Bowtie2 tool, with the exception of the microbiome metatranscriptome step [35]. The unaligned reads were assembled using an integrative strategy, which takes advantage of different tools to improve the quality and completeness of the assembled transcripts, such as SPAdes [36], rnaSPAdes [37], metaSPAdes [38], rnaviralSPAdes, metaviralSPAdes (version 3.15.5) [39], Trinity (version 2.15.1) [40], Oases (version 0.1.2) [41], and MEGAHIT (version 1.2.9) [42]. The assembled transcripts were then clustered based on 90% similarity in the CD-HIT software (version 4.8.1) [43]. 

The taxonomic assessment of the assembled transcripts > 500 nt was carried out using taxonomic classification of DIAMOND Blastx (version 2.1.1) [44] with the non-redundant proteins database (NR) and TaxonKit (version 0.14.2) [45], and plotted using R software (version 3.5.3). For further identification of viral sequences, we also applied a specific strategy based on sequence similarity searches using DIAMOND Blastx (version 2.1.1) [44] with the non-redundant proteins database (NR) and Blastn (version 2.13.0) [46] with the nucleotide database (NT). The result was filtered to select only sequences with similarity to viral sequences using in-house python scripts and reassembled using the CAP3 tool (version 10.2011) [47]. The putative viral sequences were subjected to ORF prediction using the orfipy tool (version 0.0.4) [48] with the genetic codes 1 (Standard) and 5 (Invertebrate Mitochondrial) for observation and comparison of genome structure, and analysis of conserved domains via HMMER [49] based on the Pfam database.

#### 2.3.2. Manual Curation

The sequences that showed similarity with viral sequences were submitted to Blastn and Blastx analysis via NCBI Blast Online [46] to double check the viral origin according to most updated nucleotide acid databases (Supplementary Table S3). Sequences that maintained the closest similarity to viral elements were submitted to structural annotation based on the presence of an open reading frame (ORF) using ORFfinder [50], for observation and comparison of the genome arrangement, and analysis of conserved domains via InterProScan [51].

### 2.4. Phylogenetic Analysis

Viral sequences with similarity to polymerase or polyprotein coding genes were used for phylogenetic analysis. Datasets were created consisting of public protein sequences with closely related amino acid sequences and ICTV reference sequences. Global alignment was generated for each dataset using the MAFFT online tool. The alignment was visualized and trimmed in AliView [52]. In the CIPRES Science Gateway, the edited alignment was used as input to the ModelTest program to select the best evolutionary amino acid model according to Akaike Information Criteria (AIC), and then a maximum likelihood phylogeny tree was inferred, setting 1000 bootstrap replicates. Trees were visualized using the online tool Interactive Tree of Life (ITOL v6). Bootstrap values lower than 75% are not shown.

### 2.5. Abundance of Viral Sequences

The complete transcriptome assembled for each insect species was assessed with the orfipy tool (version 0.0.4) [48] to identify the most likely ORF. Potential host transcripts were then merged with the assembled viral and host transcripts to access the viral abundance in comparison to host RNAs using the software Salmon (version 1.9.0) [53]. Host transcripts were assembled using Trinity (version 2.15.1) [40].

## 3. Results

Our search retrieved 2666 results. After manual curation, only 96 articles were used in the study. Only a small fraction (15) of the results were related to other species besides bees, which we referred to here as neglected pollinators and floral visitors. Overall, we observed only a few studies regarding insects involved in this important ecological service. This is evident from the analysis of the number of publications over the years. Before 1980, there was a significant shortage of publications related to this subject: less than 10 articles. Although there has been an increase in the number of articles, the maximum number of publications does not exceed one hundred, and many studies are still necessary to answer important questions on this matter (Supplementary Figure S1A). In terms of knowledge production by country, we found that the United States of America had the highest number of articles, totaling twenty. This was followed by Poland, Kenya, and Brazil, with the latter having seven articles (Supplementary Figure S1B). As for the plant family, we noticed a great emphasis on *Malvaceae* with 14.6%, followed by *Poaceae* with 13.5%, and *Solanaceae* with 8.3% (Supplementary Figure S1C).

The selected articles were used to construct a Sankey plot comparing *Apis mellifera* with other neglected pollinators species (Supplementary Figure S2). According to our search of the literature, four neglected pollinators were found. The species *Atrichopogon* sp., *Dasyhelea* sp., *Trigona* sp., and *Forcipomyia* sp. have been described as possible vectors of pathogens to only one plant of the family *Malvaceae*, *Theobroma cacao*, that is grown in Brazil in the states of Pará and southernmost region of Bahia. 

From the analyzed insect data, we noticed a large percentage of publications for *Apis mellifera* (85.4%) and few studies related to *Dasyhelea* (4.2%), *Trigona* (4.2%), *Forcipomyia* (5.2%), and *Atrichopogon* (1.0%) (Figure 1A). Therefore, we selected publicly available RNA-Seq libraries derived from these insects that are likely involved in pollination services but neglected regarding virome studies (Supplementary Table S2).

### 3.1. Metatranscriptome Assembly

The libraries referring to *Aphis aurantii* presented a total of 147,820,116 raw reads, distributed among six libraries with an average of 24,636,686 reads per library. The assembly step produced on average 60,271 transcripts with a maximum of 73,013 sequences assembled in the library SRR11241600 (1st instar) and a minimum of 50,559 for the sample SRR11241578 (3rd instar). Regarding *Atrichopogon* sp., the library presented a total of 20,602,753 raw reads. After assembling non-aligned sequences against the reference genome, 169,600 contigs were identified with an N50 of 625 and an average length of 417 nt. The library that was constructed from *Dasyhelea* sp. presented a total of 16,591,290 raw reads. After assembling the non-aligned sequences, 150,490 contigs were identified with an N50 of 1049 and an average length of 660 nt. The libraries referring to *Forcipomyia taiwana* presented a total of 82,132,891 raw reads, distributed among four libraries with an average of 20,533,223 per library. The assembly step produced on average 40,541 transcripts with a maximum of 70,737 sequences assembled in the library SRR13426333, and a minimum of 13,993 for the sample SRR13426334, both libraries with samples treated with 0.25 M NaCl. *Trigona ventralis hoozana* had two libraries with an average of 72,269,947 reads. After assembly, an average of 135,094 transcripts were identified, ranging from 73,463,929 (SRR5601830) to 71,075,965 (SRR5601896). The N50 varied from 2754 (SRR5601830) to 2932 (SRR5601896), with an average length of 1174 nt. The assembly information for each library can be visualized in Figure 1B and Supplementary Table S1.

### 3.2. Metagenomic Analysis

**Figure 2:**
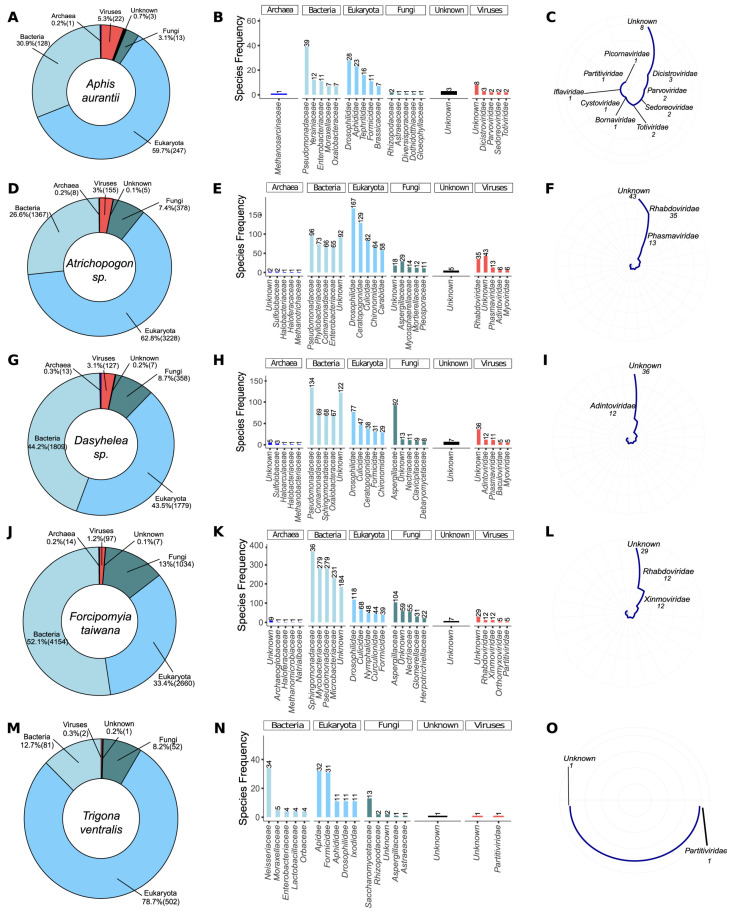
Metagenomics analysis of plant-visiting insects. The global microbiome at kingdom (**A**,**D**,**G**,**J**,**M**) and family (**B**,**E**,**H**,**K**,**N**) levels. Diversity of viral families identified in each insect (**C**,**F**,**I**,**L**,**O**).

The transcriptomic data resulting from the assembly were used for initial screening of microbial diversity using a metagenomics-based strategy with DIAMOND and TaxonKit. Eukarya (59.7%) was the most prevalent taxon group in *Aphis aurantii*, with 247 species, followed by Bacteria (128 spp., 30.9%), Fungi (13 spp., 3.1%), Viruses (22 spp., 5.3%), Unknown (3 spp., 3), and Archaea (1 spp., 0.2%) (Figure 2A). *Pseudomonadaceae* (Bacteria) was the richest family in *A. aurantii*, with 39 species, followed by *Drosophilidae* (Eukarya, 28 spp.), *Aphididae* (Eukarya, 23 species), and *Tephritidae* (Eukarya, 16 spp.) (Figure 2B). *Dicistroviridae* was the most abundant family in Viruses, with three species, followed by *Parvoviridae*, *Sedoreoviridae*, and *Totiviridae* (two species each). Unknown families were composed of eight species (Figure 2C). In *Atrichopogon* sp., Eukarya was also the richest taxon group (3228 species, 62.8%), followed by Bacteria (1367 spp., 26,6%), Fungi (378 spp., 7.4%), Viruses (155 spp., 3%), Archaea (8 spp., 0.2%), and Unknown (5 spp., 0.1%) (Figure 2D). *Drosophilidae* (Eukarya, 167 spp.), *Ceratopogonidae* (Eukarya, 129 spp.), and *Pseudomonadaceae* (Bacteria, 96 spp.) were the richest families (Figure 2E,F). Among the viruses, most of the species belonged to Unknown (45 spp.), followed by *Rhabdoviridae* (35 species) and *Phasmaviridae* (13 spp.). In *Dasyhelea* sp., we identified 1809 species coming from Bacteria (44.2%), 1779 from Eukarya (43.5%), 358 from Fungi (8.7%), 127 from Viruses (3.1%), 13 from Archaea (0.3%), and 7 from Unknown (0.2%) (Figure 2G). *Pseudomonadaceae* was the most abundant bacterial family in the sample with 134 species, followed by Unknown (Bacteria, 122 spp.) (Figure 2H). Regarding viral families, the highest abundance of transcripts was derived from elements of the Unknown families (36 spp.), followed by *Adintoviridae* (12 spp.), *Phasmaviridae* (11 sequences), *Baculoviridae*, and *Myoviridae* (5 species each) (Figure 2I). Bacteria was the most dominant taxon group in *Forcipomyia taiwana*, with 4154 species (52.1%), followed by Eukarya (2660 spp., 33.4%), Fungi (1034 spp., 13%), Viruses (97 spp., 1.2%), Archaea (14 spp., 0.2%), and Unknown (7 spp., 0.1%) (Figure 2J). In contrast to the other pollinators, *Sphingomonadaceae* (Bacteria) was the dominant family, with 366 species, followed by *Mycobacteriaceae* and *Pseudomonadaceae* (Bacteria, 279 species each), and *Microbacteriaceae* (Bacteria, 231 species) (Figure 2K). Among Viruses, most species were also within Unknown families (29 spp.) followed by *Rhabdoviridae* and *Xinmoviridae* (12 spp. each (Figure 2L)). *Trigona ventralis* has a diverse range of species belonging to different taxonomic groups. Among these, Eukarya is the richest group, with 502 species (78.7%), followed by Bacteria (81 species, 12.7%), Fungi (52 species, 8.2%), Viruses (2 species, 0.3%), and Unknown (1 species, 0.2%) (Figure 2M). *Neisseriaceae* is the most predominant family within Bacteria, with 34 species, followed by *Apidae* (Eukarya, 32 species) and *Formicidae* (Eukarya, 31 species) (Figure 2N). The two viral species identified in this study belong to *Partitiviridae* and Unknown families (Figure 2N,O). The predominant families are *Formicidae* and *Apidae*, with 32 and 30 species, respectively. The viral species identified in this case belong to different families, including *Chuviridae*, *Flaviviridae*, *Nudiviridae*, *Partitiviridae*, *Parvoviridae*, *Phasmaviridae*, and *Siphoviridae*.

### 3.3. Virome Characterization

As we identified a considerable number of viral sequences in the metagenomic analysis, we further explored the virome of plants and associated insects. Therefore, we performed regular sequence similarity searches on non-redundant NCBI databases, followed by manual inspection to remove most of the common false-positive sequences, such as retroviral and phage-related elements. Our comprehensive analysis revealed twenty-three sequences showing similarity to viral sequences, four at nucleotide (nt) level and twenty-two at protein level.

- *Aphis aurantii*

**Figure 3:**
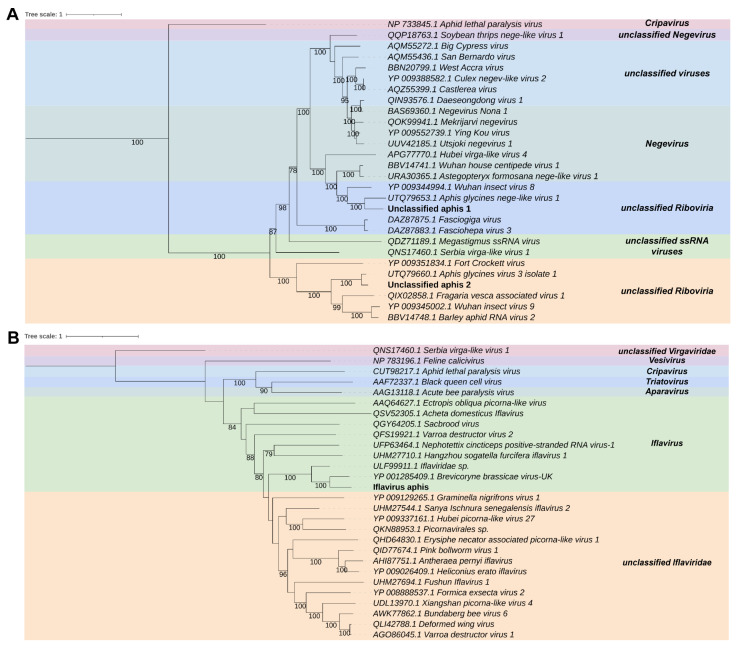
Characterization of viral sequences identified in *Aphis aurantii* samples. (**A**) Phylogenetics analyses of sequences related to members of *Riboviria*. The best model according to the Akaike information criterion (AIC) was VT + F. (**B**) Phylogeny of transcript showing similarity to members of the family *Iflaviridae*. The best model according to the Akaike information criterion (AIC) was BLOSSUM62 + F.

In the libraries of *Aphis aurantii*, we found two transcripts that share sequence similarity with unclassified elements from *Riboviria*. Aa_contig1 is a sequence that is 11,212 nt long, containing six ORFs. The first ORF is the longest, spanning 8247 nt, which is surrounded on the left by the 5′ UTR of 24 nt. The second, third, and fourth ORFs are 1284, 465, and 459 nt long, respectively. The fifth ORF overlaps with the fourth ORF and is 366 nt long, which is followed by the 3′ UTR of 83 nt. The Aa_contig1 showed similarity only at amino acid level, to the replicase protein sequence of *Aphis glycines nege-like virus 1*. The Aa_contig2 is another sequence that is 10,185 nt long encoding to four ORFs. The first ORF is 7338 nt long, preceded by a 5′ UTR of 53 nt. The second ORF of 1314 nt overlaps with the first ORF in 76 nt. The third ORF of 516 nt starts 141 nt after the end of the second ORF and is followed by the fourth ORF that contains 681 nt long succeeded by the 3′ UTR of 179 nt. This transcript also shows similarity to viruses derived from *Aphis* species at amino acid level, *Aphis glycines virus 3 isolate 1*. Aa_contig1 and Aa_contig2 contain the Alphavirus-like_MT_dom domain (IPR002588), as well as the (+)RNA_virus_helicase_core_dom (IPR027351) superimposed on the homologous superfamily P-loop_NTPase (IPR027417) in both, and Macro_dom-like (IPR043472) in Aa_contig2. Additionally, both transcripts have SP24 (IPR032441) family and the RNA-dir_pol_PSvirus (IPR007094) and Tymovirus_RNA-dep_RNA_pol (IPR001788) domains, which overlap with the DNA/RNA_pol_sf homologous superfamily (IPR043502). Aa_contig1 also has the DiSB-ORF2_chro (IPR032433) domain and Aa_contig2 also has the RNA_MeTrfase_FtsJ_dom domain (IPR002877) superimposed on the SAM-dependent_MTases_sf homologous superfamily (Supplementary Figure S3). Phylogenetic analysis shows that the putative viruses represented by the Aa_contig1 and Aa_contig2 sequences cluster with unclassified *Riboviria* sequences, closely related to *Aphis glycines nege-like virus 1* and *Aphis glycines virus 3 isolate 1*, respectively. Both *Aphis glycines nege-like virus 1* and *Aphis glycines virus 3 isolate 1* have conserved domains that are also found in Aa_contig1 and Aa_contig2 (Figure 3A). Therefore, we named these new viruses Unclassified aphis 1 (Un-ap1) and Unclassified aphis 2 (Un-ap2), respectively.

In the samples of *Aphis aurantii* we also found the Aa_contig3, which is 10,063 nt long and has a similarity to viruses of the family *Iflaviridae* at the amino acid level, specifically to the polyprotein sequence of *Brevicoryne brassicae virus—UK*. Using NCBI ORFfinder, we annotated a large ORF of 8841 nt flanked by 5′ and 3′ UTR of 830 nt and 393 nt, respectively. Aa_contig3 contained overlapping Rhv-like (IPR033703), Picornavirus_capsid (IPR001676), and Dicistrovirus_capsid-polyPr_C (IPR014872) domains superimposed on the homologous superfamily Viral_coat (IPR029053). The RNA-dir_pol_PSvirus (IPR007094) and RNA-dir_pol_C (IPR001205) domains overlapped in the Rev_trsase/Diguanyl_cyclase (IPR043128) and DNA/RNA_pol_sf (IPR043502) homologous superfamilies. Additionally, we detected the Helicase_SF3_ssRNA_vir (IPR014759) and Helicase_SF3_ssDNA/RNA_vir (IPR000605) domains, along with the homologous superfamilies Peptidase_S1_PA_chymotrypsin (IPR043504) and Peptidase_S1_PA (IPR009003) (Supplementary Figure S4). Based on phylogenetic analysis, this putative virus clustered with sequences from the genus *Iflavirus* (*Iflaviridae*) and was closely related to *Brevicoryne brassicae virus-UK* with 100% bootstrap (Figure 3B). Moreover, conserved domains in Aa_contig3 were found in the closely related *Brevicoryne brassicae picorna-like virus*. To indicate the host’s origin, this new virus was named Iflavirus aphis (Iva).

- *Atrichopogon* sp.

In *Atrichopogon* sp., we discovered five transcripts that have similarities to elements of the order *Bunyavirales*. Each transcript, labeled as A_contig4, A_contig5, A_contig6, A_contig7, and A_contig8, varies in length and contains an ORF that codes for a protein. A_contig4 is 1557 nt long containing an ORF of 1536 nt with 5′ UTR of 20 nt and an open read frame at the 3′ end. According to DIAMOND Blastx, the A_contig4 sequence shares similarity with the RNA-dependent RNA polymerase (RdRp) sequence of *Shuangao Insect Virus 3*. A_contig5 has a partial sequence with an ORF of 2268 nucleotides with open read framings at 3′ and 5′ extremities. The best DIAMOND Blastx hit for A_contig5 is the glycoprotein sequence represented by the *Wuhan mosquito virus 1*. A_contig6 is 9236 nt long, and has an ORF of 8958 nt. It has a 5′ UTR of 118 nt and a 3′ UTR of 161 nt. The best DIAMOND Blastx hit for A_contig6 is the RdRp sequence of the *Hubei diptera virus 3*. A_contig7 is 4453 nt long, and it has an ORF of 3915 nt, with a 5′ UTR of 430 nt and a 3′ UTR of 109 nt. The best DIAMOND Blastx hit for A_contig7 is the putative glycoprotein of the *Hubei diptera virus 5*. A_contig8 has 1405 nt with an ORF of 1047 nt surrounded by a 5′ UTR of 117 nt and a 3′ UTR of 242 nt. It shares similarities with the nucleocapsid sequence of the *Watermelon crinkle leaf-associated virus 1*. Regarding the presence of conserved domains, A_contig4 and A_contig6 sequences showed RNA_pol_bunyavir (IPR007322) and RNA-dir_pol_NSvirus (IPR007099) domains (Supplementary Figure S5A,C), similar to those found in the *Shuangao Insect Virus 3* and the *Hubei diptera virus 3*, respectively. However, the *Hubei diptera virus 3* has additional domains such as L_protein_N (IPR029124) and L_PA-C-like (IPR022531). A_contig7 contained Phlebovirus_G2_fusion (IPR009878) and Phlebo_G2_C (IPR043603) domains (Supplementary Figure S5D), similar to the *Hubei diptera virus 5*, which also has the Phlebovirus_G1 (IPR010826) domain. The Capsid_Phlebovir/Tenuivir (IPR009522) family was observed in A_contig8 (Supplementary Figure S5E), which was similar to the *Watermelon crinkle leaf-associated virus 1*, the best hit in amino acid searches. However, A_contig5 did not show any domains, while *Wuhan mosquito virus 1* had the Phlebovirus_G2_fusion (IPR009878) domain (Supplementary Figure S5B).

**Figure 4:**
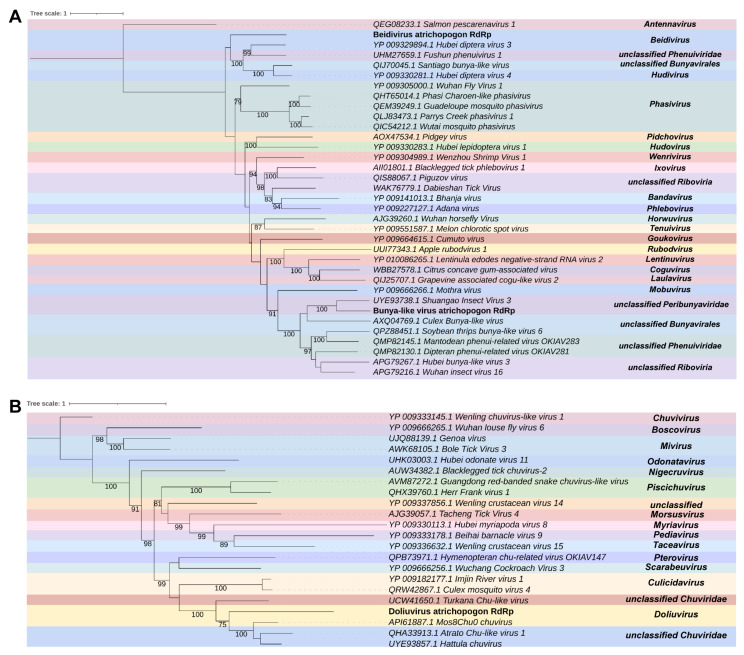
Characterization of viral sequences identified in *Atrichopogon* sp. samples. (**A**) Phylogenetics analyses of sequences related to members of the order *Bunyavirales*. The best model according to the Akaike information criterion (AIC) was VT + F. (**B**) Phylogeny of transcript showing similarity to members of the family *Chuviridae*. The best model according to the Akaike information criterion (AIC) was VT + F.

According to the phylogenetic analysis, A_contig4 and A_contig6 were found to be closely related to elements in the order *Bunyavirales*. Specifically, A_contig4 was closely related to *unclassified Peribunyaviridae* with 100% bootstrap, while A_contig6 was closely related to *Beidivirus* (*Phenuiviridae*), with 100% bootstrap. It is important to note that elements from the order *Bunyaviruses* typically have segmented genomes. Based on the analyzed transcripts, it appears that A_contig4 and A_contig5 are probably the RdRP and glycoprotein segments of the same putative virus in *unclassified Peribunyaviridae*, respectively. On the other hand, A_contig6, A_contig7, and A_contig8 are probably the segments of the same putative virus represented by the *Beidivirus* (*Phenuiviridae*), with A_contig6 as the RdRp segment, A_contig7 as the glycoprotein segment, and A_contig8 as the nucleocapsid segment (Figure 4A). These new viruses were named Bunya-like virus atrichopogon (BlVa) and Beidivirus atrichopogon (BVa).

We found two transcripts in the library that resemble elements of the family *Chuviridae*. The first transcript, A_contig9, has 1680 nt and contains an incomplete ORF, with open read framings at 3′ and 5′ extremities. This transcript shows similarity to the *Atrato Chu-like virus 1* RdRp sequence, according to the DIAMOND Blastx hit. The second transcript, A_contig10, has 4789 nt and contains two ORFs, of 2013 nt and 1485 nt, respectively, surrounded by a 5′ UTR of 641 nt and 3′ UTR of 564 nt. This transcript shares similarity with the segment G-N (containing Glycoprotein and Nucleocapsid) of the *Mos8Chu0 chuvirus*, according to DIAMOND Blastx hit. However, there were no conserved domains for either of these contigs (Supplementary Figure S6). Only the *Atrato Chu-like virus 1* RdRp sequence presented the conserved domains Mononeg_mRNAcap (IPR026890) and Mononeg_RNA_pol_cat (IPR014023), although A_contig9 did not present any conserved domains. Phylogenetic analysis revealed that A_contig9 was closely related to elements of the genus *Doliuvirus* (*Chuviridae*) (Figure 4B). This suggests that A_contig9 and A_contig10 are likely putative RdPp and glycoprotein-nucleocapsid sequences of the same virus. We named this new virus Doliuvirus atrichopogon (DVa).

- *Dasyhelea* sp.

**Figure 5:**
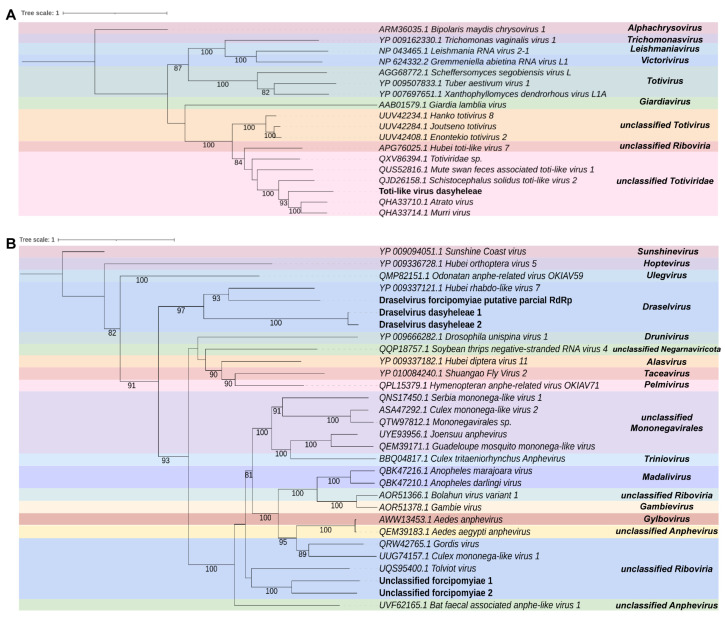
Characterization of viral sequences identified in *Dasyhelea* sp. samples. (**A**) Phylogenetics analyses of sequences related to members of the family *Totiviridae*. The best model according to the Akaike information criterion (AIC) was BLOSSUM62 + F. (**B**) Phylogeny of transcript showing similarity to members of the family *Xinmoviridae*. The best model according to the Akaike information criterion (AIC) was VT + F.

The transcript D_contig11 of 6797 nt identified in the *Dasyhelea* sp. library showed similarity to the RdRp sequence of *Atrato virus*, which belongs to the family *Totiviridae*. The NCBI ORFfinder tool revealed two large ORFs containing 3063 nt and 2981 nt surrounded by the 5′ and 3′ UTRs of 536 nt and 80 nt, respectively. For one of the ORFs from D_contig11, conserved domains were detected, including the RNA-dir_pol_luteovirus family (IPR001795) and the homologous superfamily DNA/RNA_pol_sf (IPR043502) (Supplementary Figure S7). Phylogenetic analysis showed that the putative virus clustered with sequences from the family *unclassified Totiviridae*, closely related to the group that contains the *Atrato virus*, with 93% bootstrap (Figure 5A). The conserved domains found in D_contig11 were also identified in the closely related *Atrato virus*. Based on these findings, this new virus has been named Toti-like virus dasyheleae (TlVd).

We also found in the *Dasyhelea* sp. library two transcripts that shared similarities with elements of the family *Xinmoviridae*. The first transcript, D_contig12, was 3812 nt long and had an ORF of 3360 nt, with a 5′ untranslated region (UTR) of 451 nt and an open read framing at 3′ extremity. The second transcript, D_contig13, was 4803 nt long, presented 4 ORFs, the largest being 1995 nt long and surrounded by a 5′ UTR of less than 684 nt. The following ORFs have 423, 399 and 468 nt, respectively, with a 3′ UTR of 41 nt. Both transcripts were similar to the RNA-dependent RNA polymerase (RdRp) sequence of *Hubei rhabdo-like virus 7*, according to the DIAMOND Blastx best hit. We found that both transcripts shared the Mononeg_RNA_pol_cat domain (IPR014023), although they do not show similarity at nucleotide level between themselves (Supplementary Figure S8). When compared to the closely related virus, the *Hubei rhabdo-like virus 7*, we observed that they presented the similar domains, except for three domains that were absent in the non-retroviral contigs of our study. These domains were RNA-dir_pol_paramyxovirus (IPR016269), Mononeg_mRNAcap (IPR026890), and Mononega_L_MeTrphase (IPR025786). Phylogenetic analysis revealed that both transcripts were closely related to elements of the genus *Draselvirus* (*Xinmoviridae*) (Figure 5B). We named these new viruses Draselvirus dasyheleae 1 (DVd1) and Draselvirus dasyheleae 2 (DVd2).

- *Forcipomyia taiwana*

In the *Forcipomyia taiwana* libraries, we found four transcripts that were similar to elements of the family *Xinmoviridae*. Ft_contig14 is a sequence that is 12,795 nt long containing three ORFs. The first ORF spanning 1665 nt is surrounded on the left by the 5′ UTR of 218 nt. The second ORF is 2526 nt long, and the third is the longest ORF, presenting 6402 nt, which is followed by the 3′ UTR of 541 nt. The Ft_contig14 showed similarity only at amino acid level to the RdRp sequence of the *Aedes albopictus anphevirus*. Ft_contig15 was 6687 nt long, with an ORF of 6270 nt. It has a 5′ UTR of 409 nt and a 3′ UTR of 209 nt, similar to the RdRp sequence of *Tolviot virus*. Ft_contig16 and Ft_contig17 were similar to the RdRp sequence of the *Hubei rhabdo-like virus 7* probably representing fragments of the same virus. Ft_contig16 was 1038 nt long, with an ORF of 990 nt. It had a 5′ UTR of 49 nt and open reading frame at 3′ UTR. Ft_contig17 was 855 nt long, with an ORF of 705 nt. It had a 5′ UTR of 151 nt and also showed an open reading frame at 3′ UTR. All of these sequences, except Ft_contig16 (Supplementary Figure S9C), showed the Mononeg_mRNAcap (IPR026890) domain. Ft_contig14 and the *Aedes albopictus anphevirus* had the first three domains, but not RNA-dir_pol_paramyxovirus (IPR016269) (Supplementary Figure S9A). Ft_contig15 and the *Tolviot virus* had Mononeg_RNA_pol_cat (IPR014023) and Mononega_L_MeTrfase (IPR025786) (Supplementary Figure S9B). Ft_contig17 only showed Mononeg_mRNAcap (IPR026890) (Supplementary Figure S9D). And the *Hubei rhabdo-like virus 7* had additional domains, including Mononeg_RNA_pol_cat (IPR014023), Mononega_L_MeTrfase (IPR025786), and RNA-dir_pol_paramyxovirus (IPR016269) families. Phylogenetic analysis revealed that Ft_contig14, Ft_contig15, Ft_contig16, and Ft_contig17 were closely related to elements of the genus *Draselvius* (*Xinmoviridae*) and *Unclassified* (Figure 5B). We named these new viruses Unclassified forcipomyiae 1 (Un-f1), Unclassified forcipomyiae 2 (Un-f2), and Draselvirus forcipomyiae (DVf-RdRp), respectively. We also found a transcript that is similar to the putative glycoprotein sequence of the *Atrato Rhabdo-like virus 2*, and it is probably the glycoprotein sequence of the new virus Draselvirus forcipomyiae putative glycoprotein (DVf-PGp). This transcript is called Ft_contig18 and has a length of 6208 nt, containing four ORFs. The first ORF spanning 1407 nt is surrounded on the left by the 5′ UTR of 181 nt. The second and third ORFs are 1104 and 825 nt long, respectively. The fourth is the longest ORF and is 1983 nt long, which is followed by the 3′ UTR of 411 nt. Regarding the presence of conserved domains, only the family Rhabdo_ncap_2 (IPR004902) was detected in Ft_contig18 (Supplementary Figure S9E), while the *Atrato Rhabdo-like virus 2* exhibited both Mononeg_mRNAcap (IPR026890) and Mononeg_RNA_pol_cat (IPR014023) domains.

**Figure 6:**
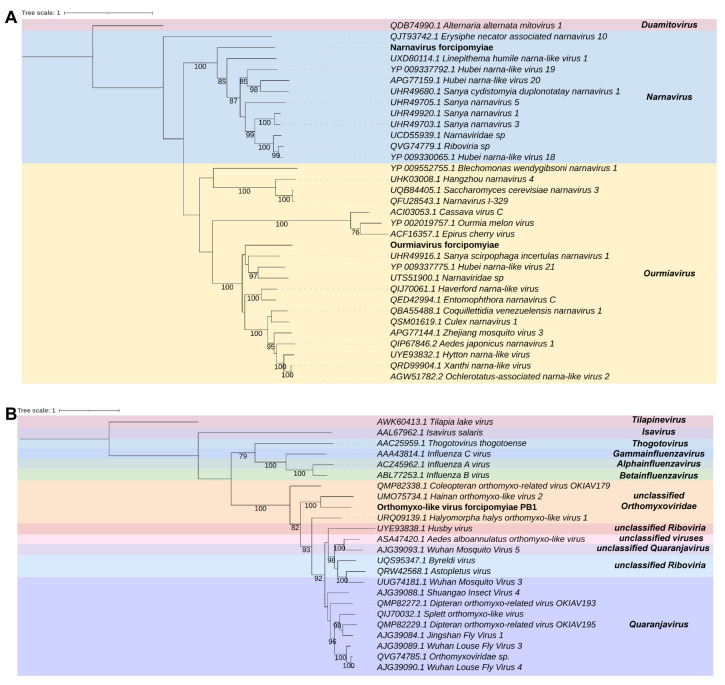
Characterization of viral sequences identified in samples of *Forcipomyia taiwana*. (**A**) Phylogenetics analyses of sequences related to members of the families *Narnaviridae* and *Ourmiaviridae*. The best model according to the Akaike information criterion (AIC) was BLOSSUM62 + F. (**B**) Phylogeny of transcript showing similarity to members of the family *Orthomyxoviridae*. The best model according to the Akaike information criterion (AIC) was BLOSSUM62 + F.

Two additional transcripts were identified in the libraries, which were similar in sequence to elements of the families *Narnaviridae* and *Ourmiaviridae*. The first transcript, Ft_contig19, has 2981 nt with an ORF of 2934 nt with 5′ and 3′ UTRs of 34 nt and 14 nt, respectively. It displayed similarity to the RdRp sequence of the *Entomophthora narnavirus C*. The second transcript, Ft_contig20, is 2285 nt long with an ORF of 2226 nt surrounded by 5′ and 3′ UTRs of 27 nt and 33 nt, respectively. It has similarity to the RdRp sequence of the *Hubei narna-like virus 18*. Both transcripts showed the DNA/RNA_pol_sf (IPR043502) homologous superfamily (Supplementary Figure S10). The *Hubei narna-like virus 18* also presented the RNA-dir_Rpol_cat_phage (IPR007096) domain, RNA_pol_mitovir (IPR008686) family, and DNA/RNA_pol_sf (IPR043502) homologous superfamily, while the *Entomophthora narnavirus C* did not show any domains. Based on the phylogeny of the putative viruses (Figure 6A), these new viruses were classified as *Narnavirus* and *Ourmiavirus* and were named Narnavirus forcipomyiae (NVf) and Ourmiavirus forcipomyiae (OVf), respectively.

We also found two more transcripts in the libraries that are similar to elements of the family *Orthomyxoviridae*. Ft_contig21 has a length of 2478 nt and an ORF of 2433 nt surrounded by a 5′ and 3′ UTR of 31 nt and 15 nt, respectively. It is similar to the PB1 sequence of the *Hainan orthomyxo-like virus 2*. Ft_contig22 has a length of 2253 nt and an ORF of 2202 nt surrounded by a 5′ of 52 nt and an open read frame at the 3′ end. It is similar to the polymerase PA sequence of the *Hainan orthomyxo-like virus 2*. Ft_contig21 and the *Hainan orthomyxo-like virus 2* both shared the RNA-dir_pol_NSvirus (IPR007099) domain and the RNA_pol_PB1_influenza (IPR001407) family, while Ft_contig22 shared the PA/PA-X_sf (IPR038372) homologous superfamily (Supplementary Figure S11). The phylogeny of the putative virus shows that Ft_contig21 is closely related to elements of the family *Orthomyxoviridae* (Figure 6B). This virus was named Orthomyxo-like virus forcipomyiae (OlVf).

### 3.4. Abundance of Viral Sequences

**Figure 7:**
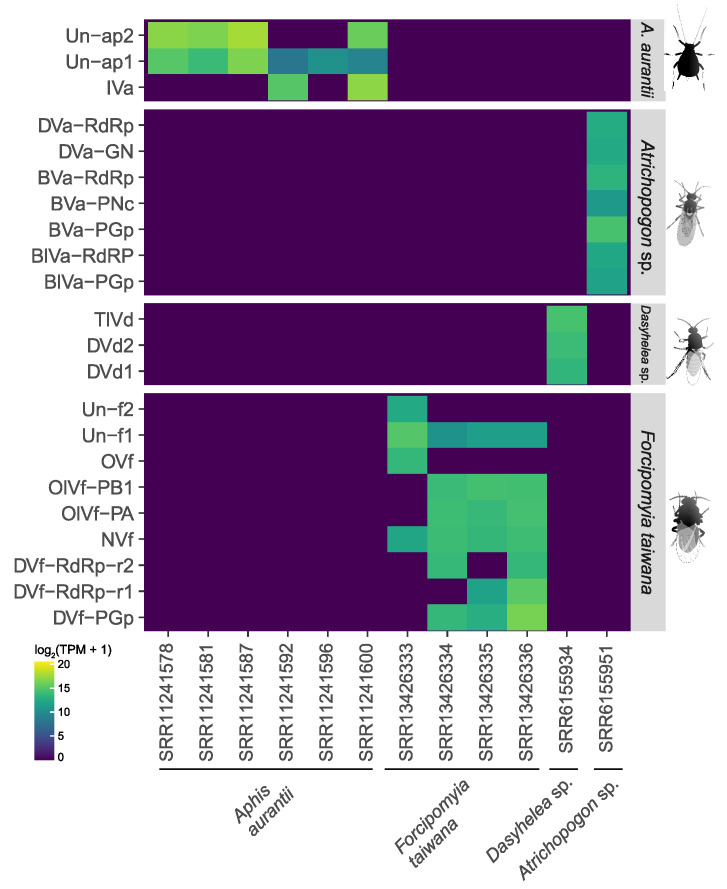
Assessment of the transcriptional activity of virus-derived sequences. Abundance of transcripts was normalized by Transcripts Per Million (TPM).

When examining the abundance of viral transcripts in *A. aurantii*, each library representing a distinct developmental stage of the organism was quantified separately. These stages included 1st instar nymph (SRR11241600), 2nd instar nymph (SRR11241581), 3rd instar nymph (SRR11241578), 4th instar nymph (SRR11241596), adult without wings (SRR11241592), and adult with wings (SRR11241587). Un-ap2 and Un-ap1 transcripts had the highest abundance in most stages, except for 1st instar nymph. Un-ap1 had values ranging from 0.003 in the adult without wings to 17 in the adult with wings, while Un-ap1 had TPM values ranging from 0 in the adult without wings and 4th instar nymph to 54 in the adult with wings. IVa was only present in the 1st instar (TPM = 25) and in the adult without wings (TPM = 3.5). In summary, the transcriptional activity of Un-ap1 and Un-ap2 was highest in the adult with wings and lowest in the adult without wings, while IVa had the highest values in 1st instar nymph and in the wingless adult (Figure 7).

BVa-PGp had the highest transcriptional activity in *Atrichopogon* sp. (SRR6155951), with TPM = 2.1 (Figure 7). The quantification of viral sequences indicated low transcriptional activity for most of the virus sequences, with 0.06 for BVa-PNc to 0.58 for BVa-RdRp. The quantification of viral sequences in *Dasyhelea* sp. indicated low transcriptional activity of TlVd, DVd2, and DVd1 transcripts, with TPM values of 2.2, 1.2, and 0.71, respectively. The abundance of viral transcripts in *F. taiwana* eggs was examined separately for each library representing a distinct treatment, which included treatment with freshwater (SRR13426335 and SRR13426336) and with 0.25 M NaCl (SRR13426333 and SRR13426334). DVf-PGp (TPM = 12.5) had the highest abundance in freshwater treatments. In the 0.25 M NaCl treatment, which was used to wash away microorganisms from the eggshell, all the viruses showed lower transcriptional activity (Figure 7).

## 4. Discussion

Most vectors of animal/human pathogens are generalists regarding their intermediate or final hosts. However, this relationship tends to be more specific within plant systems, as evidenced by *Aphis craccivora*, a vector of numerous plant viruses. It has also been discovered that most vectors of plant pathogens belong to the order Hemiptera [54]. Moreover, many studies reported the importance of pollinator species in plant development and reproduction [55]. However, the majority of the studies usually describe only *Apis mellifera* and other species from the genus *Apis* helping in the pollination process [56]. In our study, we aimed to investigate the virome of different pollinators, including species that are rarely described in the literature. According to our results, four species are described as plant pollinators, but are found mainly in one species of the family *Malvaceae*. This result may demonstrate that some pollinators have a certain tropism for some plant species, or have not yet been described in the pollination process of other plant families. Additionally, this result made us question whether the virome of these species would be similar or not.

Analyzing public libraries, we identified 16 putative viral sequences from different families. This result demonstrates high viral diversity in these insects and also suggests that they may interfere in the pollination process or could be plant pathogens.

*A. aurantii*, *Atrichopogon* sp. And *F. taiwana* were the only host species that presented viruses closely related to species already known for infecting plants and other insects and causing diseases, although *A. aurantii* was the only species with high TPM values for its viruses. *T. ventralis hoozana* did not present any novel virus. Two of the viruses discovered in *A. aurantii* (Un-ap1 and Un-ap2) were closely related to Aphid viruses of the *unclassified Riboviria* (*Aphis glycines nege-like virus 1* and *Aphis glycines virus 3 isolate 1*), while the other (Iva) were clustered with a member of the genus *Iflavirus* (*Brevicoryne brassicae virus-UK*). These viruses are known as aphid pathogenic RNA viruses, a group of aphid-infecting viruses already related to dramatic negative impacts on the growth, fecundity, development, dispersal, and functions of aphid species, such as the *Aphid lethal paralysis virus* (ALPV), the *Myzus persicae densovirus* (MpDV), and the *Rhopalosiphum padi virus* (RhPV) [57]. Despite our limited knowledge of the infection mechanisms, impact, and transmission of the taxonomic groups to which the new viruses belong, there is a possibility that Un-ap2 and Un-ap1 could be affecting *A. aurantii*, particularly during the 3rd instar nymph stage. To counteract any negative effects caused by high transcriptional activity, the virus may even be affecting the vector’s physiology, which could impact the likelihood of transmission, as observed in the *Rice stripe virus* (RSV) infecting *Laodelphax striatellus* [58]. However, it is critical to understand how these new viruses interact with aphids, host plants, and non-target insects to determine their potential for disease incidence, particularly in *T. cacao* plantations, since our search of the literature returned that most of these floral visitors were described as associated only with cocoa plants. Additionally, it is essential to gain a comprehensive understanding of how virus replication can interfere with the replication of other viruses and how these interactions can impact the host. It was observed that the transcriptional activity of Un-ap1 and Un-ap2 is seemingly influenced by the low transcriptional activity of IVa, and vice versa, as evident in the comparison between adults with and without wings.

Beidivirus atrichopogon found in *Atrichopogon* sp. was phylogenetically related to the family *Phenuivirade*, which consists of enveloped, spherical, 2–8 segmented negative-stranded RNA ((-)ssRNA) viruses with diameters ranging from 80 to 120 nm that can infect three kingdoms of host organisms (animals, plants, and fungi) [59]. It is likely that this new virus has three segments consisting of an RdRp, a glycoprotein, and a nucleocapsid, as it shows similarity to and is phylogenetically closely related to the RdRp sequence of the *Hubei diptera virus 3*. *Phenuivirade* is known for comprehending various virus genera related to plant diseases, such as *Tenuivirus* and *Coguvirus*, and have different viral components associated with insect transmission and plant infection [59,60,61]. Although the exact mechanism by which plant viruses cause symptoms in their hosts is not completely known, it has been demonstrated that this family, particularly the genus *Tenuivirus*, can interfere with vital plant function, such as host cell replication, translation machinery, and ion homeostasis. For instance, the *Rice grassy stunt virus* (RGSV) protein p5, found in RNA segment 5, can disrupt the CBL-CIPK Ca^2+^ signaling network, which plays a role in regulating ion homeostasis in the plant. This can lead to a decrease in potassium content, resulting in a potassium deficit. Additionally, the protein p3 of the same virus can cause the production of an E3 ubiquitin ligase that leads to the breakdown of one subunit of plant-specific RNA polymerase IV, which is essential for RNA-directed DNA methylation [56,58]. *Phenuiviridae* viruses have already been shown being transmitted through horizontal and vertical transmission in insects and they can be transmitted to plants through salivary glands [59,60,61]. This demands the understanding on how these new viruses can be transmitted to other plants and if they can cause any diseases in the plant species, although no substantial TPM values were found. A new virus called Doliuvirus atrichopogon was also discovered in *Atrichopogon* sp. This virus is closely related to the family *Chuviridae*, which is made up of segmented negative-stranded RNA viruses infecting a wide variety of invertebrate hosts [62]. 

Another virus, Orthomyxo-like virus forcipomyiae, was found in *Forcipomyia taiwana* and is related to the family *Orthomyxoviridae*. This family also consists of (-)ssRNA viruses, but with 6–8 segments and three different RNA polymerases (polymerase acidic protein-PA, polymerase basic protein 1-PB1, polymerase basic protein 2-PB2) [62]. *Orthomyxoviridae* viruses also mainly infect mammals and are linked to various diseases, including influenza (such as *Alphainfluenzavirus*, *Betainfluenzavirus*, *Deltainfluenzavirus*, and *Gammainfluenzavirus*) [63] and tick-borne diseases. However, there are no reports of either of these viruses infecting plants or causing any diseases in pollinators.

Two viruses related to pathogenic families *Narnaviridae* and *Ourmiaviridae* were also identified in *F. taiwana*. *Narnaviridae* is characterized by a single molecule of non-encapsidated (+)ssRNA virus of 2.3–2.9 kb that encodes a single protein of 80–104 kDa with amino acid sequence motifs that are typical of an RNA dependent RNA polymerase (RdRp) [64]. The study found that NVf was phylogenetically related to organisms of the family *Narnaviridae*, such as the *Hubei narna-like virus 18*, which includes information related to the homologous superfamily DNA/RNA_pol_sf. Narnaviruses have been found in plant-pathogenic fungi, as in the study by Lin et al. (2020) [65] who detected *Magnaporthe oryzae narnavirus virus 1* (MoNV1) in the rice blast fungus *Magnaporthe oryzae* [65]. Additionally, research by Fonseca et al. (2021) [66] has shown that narnaviruses are primarily identified in insects and other arthropods.

The family *Ourmiaviridae* is known for its unique fine structure, which consists of a series of discrete lengths ranging from 30 to 62 nm. It is noteworthy that the RdRp of this family has the closest similarity to the genus *Narnavirus* (*Narnaviridae*) [67]. The origin of this family is believed to be due to a narnavirus that lacked a capsid and used to infect pathogenic fungus or endophyte, which later became a plant virus [68]. Some studies have identified members of the family *Ourmiaviridae* infecting the cytoplasm of filamentous fungi, as noted by Fonseca and collaborators (2021) [66]. Research conducted by Rastgou et al. (2009) [68] revealed that OuMV, a member of the family *Ourmiaviridae*, is responsible for causing mosaic disease in melons (*Cucumis melo*) in Iran. In their study, they discussed the phylogenetic relationships between OuMV and two other RNA viruses, as well as the implications for the evolutionary significance and taxonomic position of the genus *Ourmiavirus*. Although there are not many studies citing *Entomophthora narnavirus C*, it was detected in association with insect-associated viral contigs from RNA sequencing data from samples of *Thrips tabaci* by Chiapello et al. (2021) [69].

*Dasyhelea* sp. presented three new viruses, with the first one (TlVd) belonging to unclassified *Totiviridae* and the other two (DVd1 and DVd2) being phylogenetically closely related to the genus *Draselvirus* (*Xinmoviridae*). *Totiviridae* virions are non-enveloped icosahedral virions measuring approximately 40 nanometers in diameter, with a monopartite double-stranded RNA (dsRNA) genome ranging in size from 4.6 to 7.0 kilobase pairs. The genomes are typically organized into two ORFs, with ORF1 encoding a 70–100 kilodaltons capsid protein and ORF2 encoding an RNA-dependent RNA polymerase (RdRp). The replication takes place in the cytoplasm [70]. TlVd was related to and shared the RNA-dir pol luteovirus family (IPR001795) and the DNA/RNA pol sf (IPR043502) domains with *Atrato virus*, which is not typically associated with insect or plant diseases. The other two viruses were related to the family *Xinmoviridae*, which has single genomes segment of ~12 kb in length that encodes six/seven open reading frames (ORFs) [71]. *Aedes albopictus anphevirus* (AealbAV), which has demonstrated the being targeted by the host interference (RNAi) response with persistent virus replication [72], is one of many *Xinmoviridae* viruses that have already been registered for mosquitoes, despite the lack of information regarding their pathogenicity.

In our study, the data provided suggest the presence of diverse insect and plant-infecting viruses that could potentially compromise the health and survival of pollinators and floral visitors, as well as other important commercial plant species. There has been no specific research on the effect of ceratopogonids on native plants pollination or their degree of insect–plant specificity, but it is possible to infer that a reduction in their diversity could alter pollination rates in some plant species [73]. It is also noteworthy that the aphids penetrate the plant cuticle and insert the stylet into the intracellular space, targeting the sieve tubes of the phloem. Following that, they secrete saliva and ingest sap alternately, which may result in the exchange of microorganisms, including viruses, between the insect and the plant [74]. Therefore, further studies are needed to fully characterize the sixteen transcripts described here in order to know whether the viral transcripts infect plants with economic value. It is important to highlight that the only virus previously described in *A. aurantii* was the *Citrus Tristeza Virus* [16]. Therefore, it is critical to assess the potential pathogenicity of these viruses to pollinators and in order to develop appropriate management and mitigation strategies for both plant and insect populations, also taking into account the viral infection in different insect developmental stages. This requires additional experiments to confirm the presence of these actual viruses, which include filling sequence gaps in order to have a complete genome of the virus, testing for virus replication, visualizing virus particles, virus isolation, and testing virus pathogenicity in insects and plants. The significance of the virus-derived sequences discovered in this study is not diminished by virus validation. However, it is crucial to grasp the true extent of the virome diversity within pollinators. The mere discovery of a virus-derived sequence does not automatically imply its origin from insect-infecting viruses; instead, it could emanate from associated organisms, ingested materials, or even virus-derived endogenous viral elements (EVEs) [75].

## Data Availability

The datasets generated and analyzed during the current study are available in the DDBJ/ENA/GenBank databases under the accession numbers TPA: BK063242-BK063263.

## Supplementary Materials

The following supporting information can be downloaded at: https://www.mdpi.com/article/10.3390/v15091850/s1. Table S1: Literature mining regarding floral-visiting insects. Table S2: Overview of publicly available RNA-Seq libraries used in the study. Table S3: Closest hits to the identified viral sequences based on sequence similarity search at nucleotide and amino acid levels. Figure S1. Overview of public data on plants and associated insects of publications on plants and associated insects from 1960 to 2023, geographical distribution, and plant families. Figure S2. Sankey plot with overview of public data on species of pollinators and floral visitors associated with plants. Figure S3. Transcripts related to members of *Riboviria*. Representation with the ORF and domains. Figure S4. Transcripts related to members of the family *Iflaviridae*. Representation with the ORF and domains. Figure S5. Transcripts related to members of the order *Bunyavirales*. Representation with the ORF and domains. Figure S6. Transcripts related to members of the family *Chuviridae*. Representation with the ORF and domains. Figure S7. Transcripts related to members of the family *Totiviridae*. Representation with the ORF and domains. Figure S8. Transcripts related to members of the family *Xinmoviridae*. Representation with the ORF and domains. Figure S9. Transcripts related to members of the family *Xinmoviridae*. Representation with the ORF and domains. Figure S10. Transcripts related to members of the families *Narnaviridae* and *Ourmiaviridae*. Representation with the ORF and domains. Figure S11. Transcripts related to members of the family *Orthomyxoviridae*. Representation with the ORF and domains.
