# Supplementary material for: Mining Public Data to Investigate the Virome of Neglected Pollinators and Other Floral Visitors
Source: Viruses. 2023 Aug 31;15(9):1850. doi: 10.3390/v15091850 (PMC10535300; doi:10.3390/v15091850)
Supplement: Supplementary file 1 [file viruses-15-01850-s001.zip › de Santana et al, Supplementary Information - revision 2-prior submission.pdf]

SUPPLEMENTARY INFORMATION

Mining public data to investigate the virome of neglected pollinators and other floral visitors

Sabrina Ferreira de Santana[1†]; Vinícius Castro Santos[2†]; Ícaro Santos Lopes[2†]; Joel Augusto Moura Porto[1]; Irma Yuliana Mora Ocampo[1]; George Andrade Sodré[1]; Carlos Priminho Pirovani[1]; Aristóteles Góes Neto[2]; Luis Gustavo Carvalho Pacheco[4]; Paula Luize Camargos Fonseca[1,5]; Marco Antônio Costa[1]; Eric Roberto Guimarães Rocha Aguiar[1*]

[1] Department of Biological Science, Center of Biotechnology and Genetics, Universidade Estadual de Santa Cruz, Ilhéus, Bahia, 45662-900, Brazil; sfsantana@uesc.br (S.F.d.S.)

[2] Department of Biochemistry and Immunology, Universidade Federal de Minas Gerais, Belo Horizonte 31270-901, Brazil

[3] Department of Microbiology, Institute of Biological Sciences, Federal University of Minas Gerais, Belo Horizonte, Minas Gerais, 31270-901, Brazil

[4] Department of Biotechnology, Institute of Health Sciences, Universidade Federal da Bahia, Bahia, 40231-300, Brazil

[5] Department of Genetic, Institute of Biological Sciences, Universidade Federal de Minas Gerais, Belo Horizonte, Minas Gerais, 31270-901, Brazil

* Correspondence: ericgdp@gmail.com

† These authors contributed equally to this work.

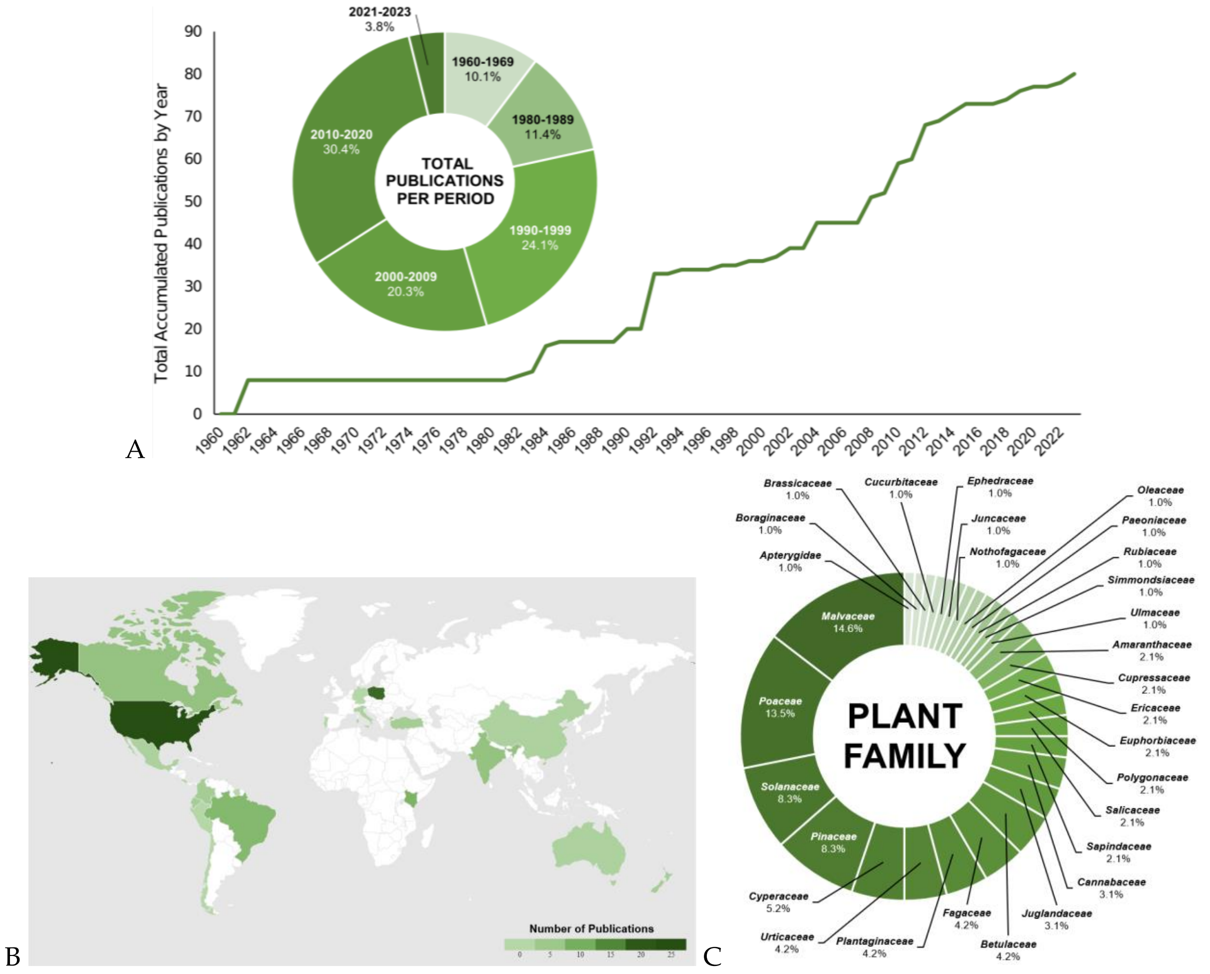

Supplementary Figure S1. Overview of public data on plants and associated insects (A) of publications on plants and associated insects from 1960 to 2023, (B) of geographical distribution and (C) of plant families.

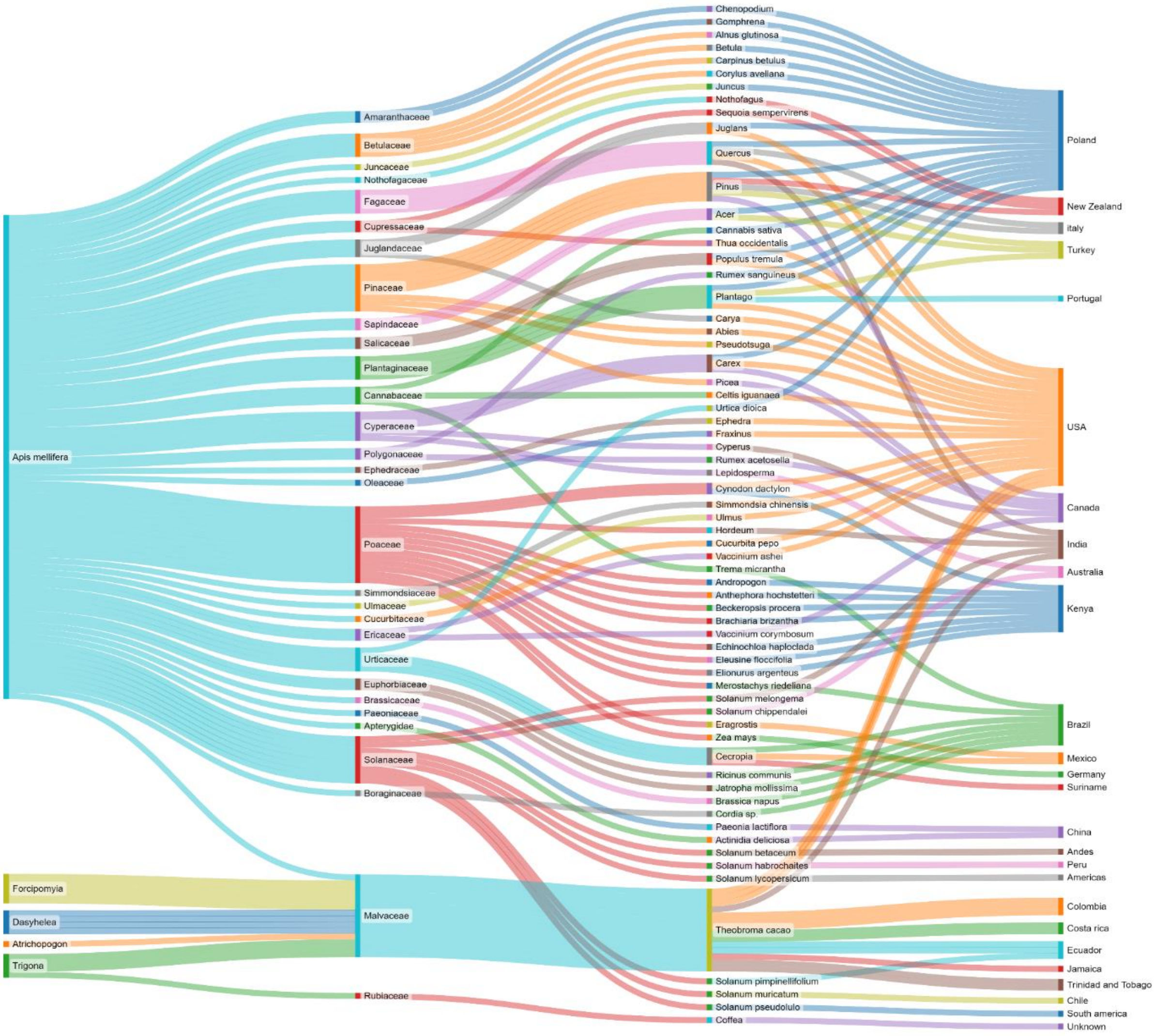

Supplementary Figure S2. Sankey plot with overview of public data on species of pollinators and floral visitors associated with plants.

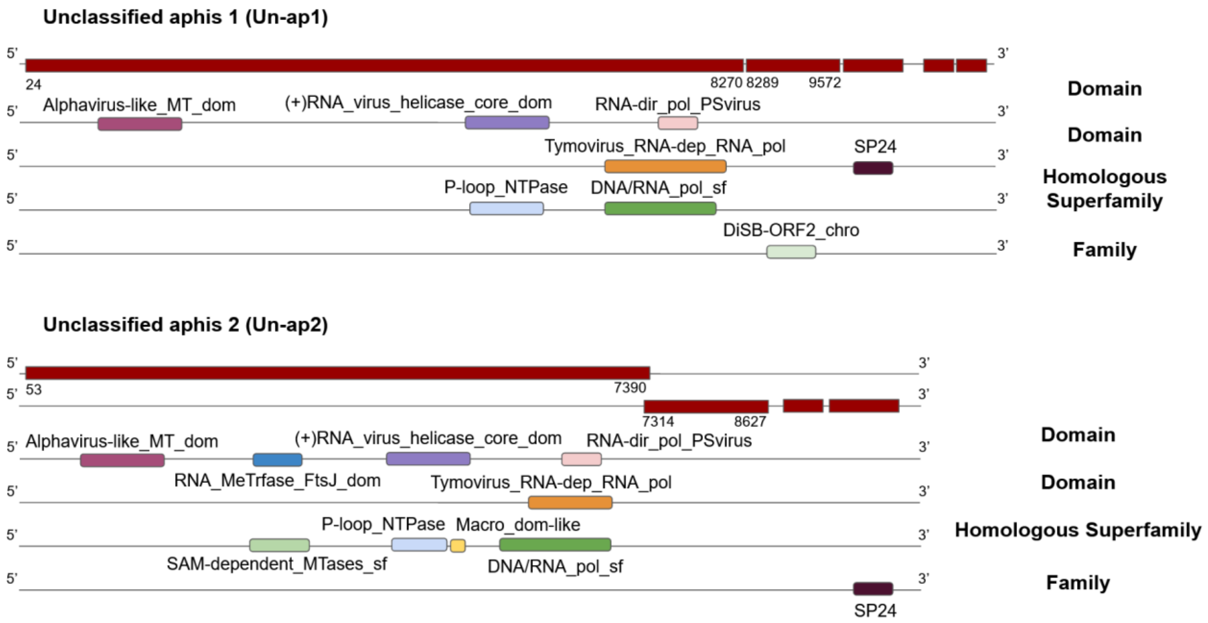

Supplementary Figure S3. Transcripts related to members of *Riboviria*. Representation with the ORF and domains.

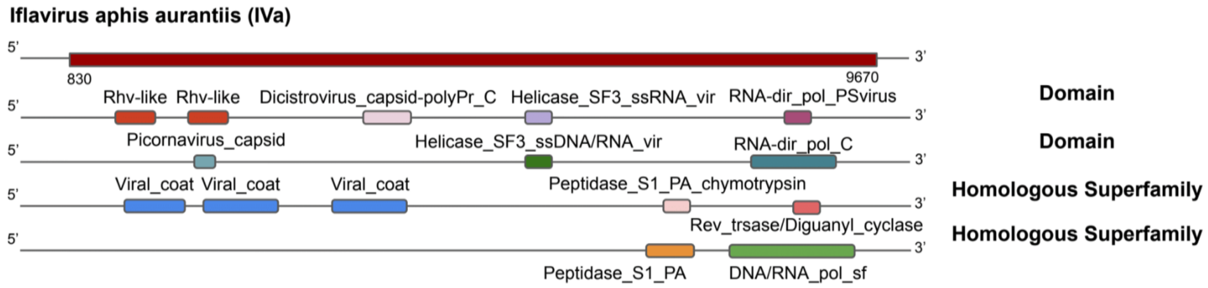

Supplementary Figure S4. Transcripts related to members of the family *Iflaviridae*. Representation with the ORF and domains.

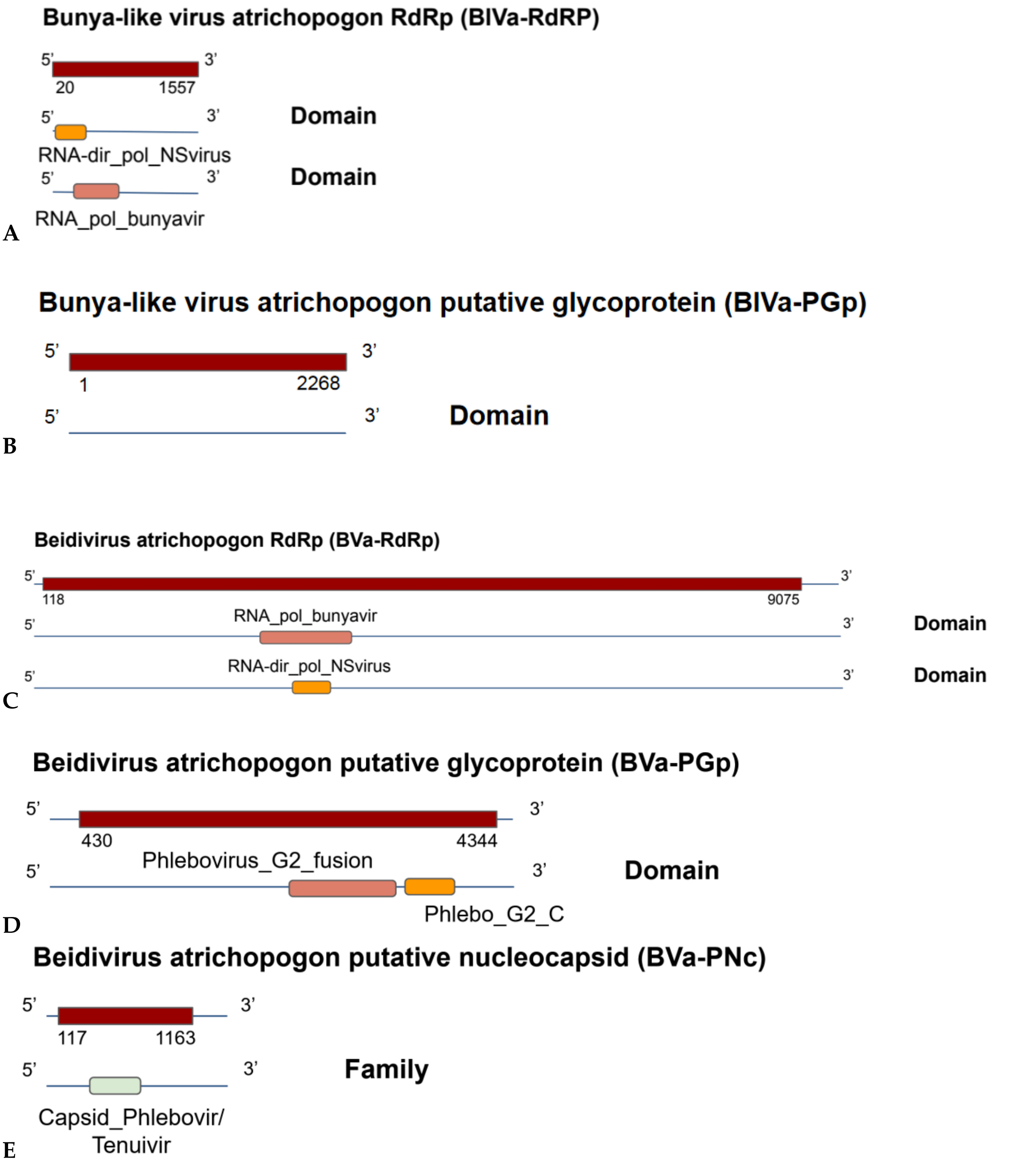

Supplementary Figure S5. Transcripts related to members of the order *Bunyavirales*. Representation with the ORF and domains.

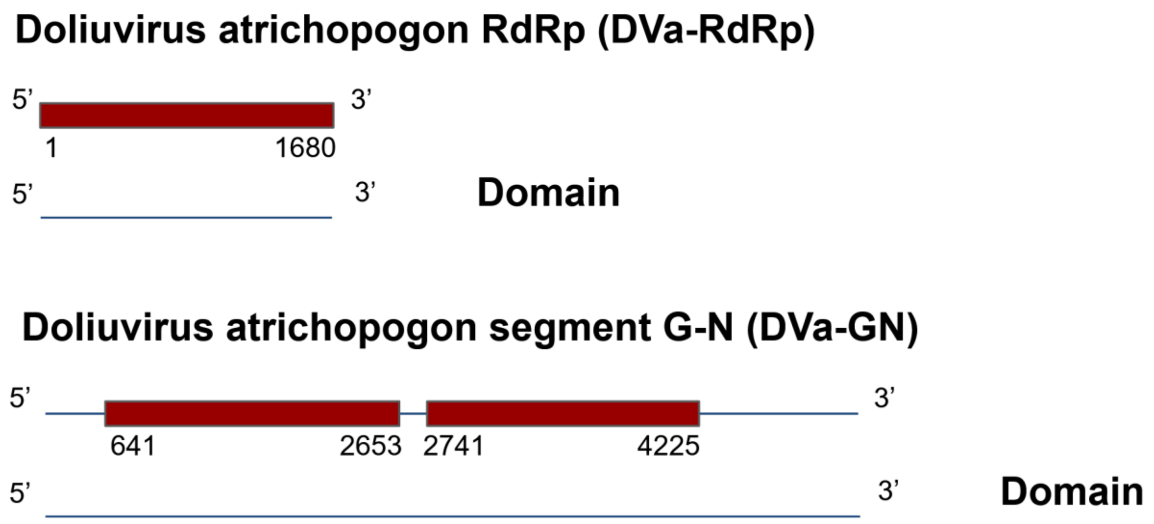

Supplementary Figure S6. Transcripts related to members of the family *Chuviridae*. Representation with the ORF and domains.

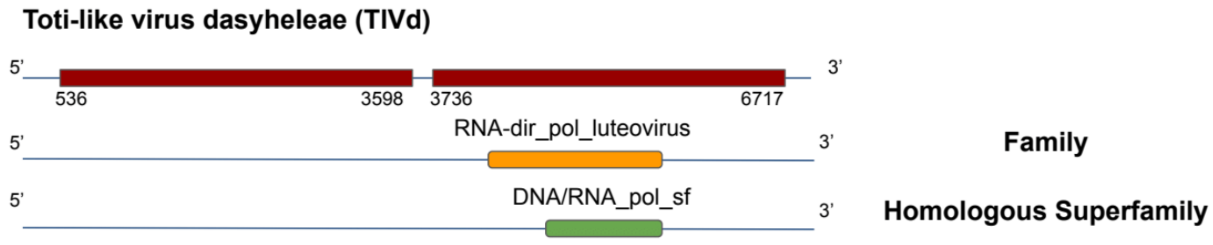

Supplementary Figure S7. Transcripts related to members of the family *Totiviridae*. Representation with the ORF and domains.

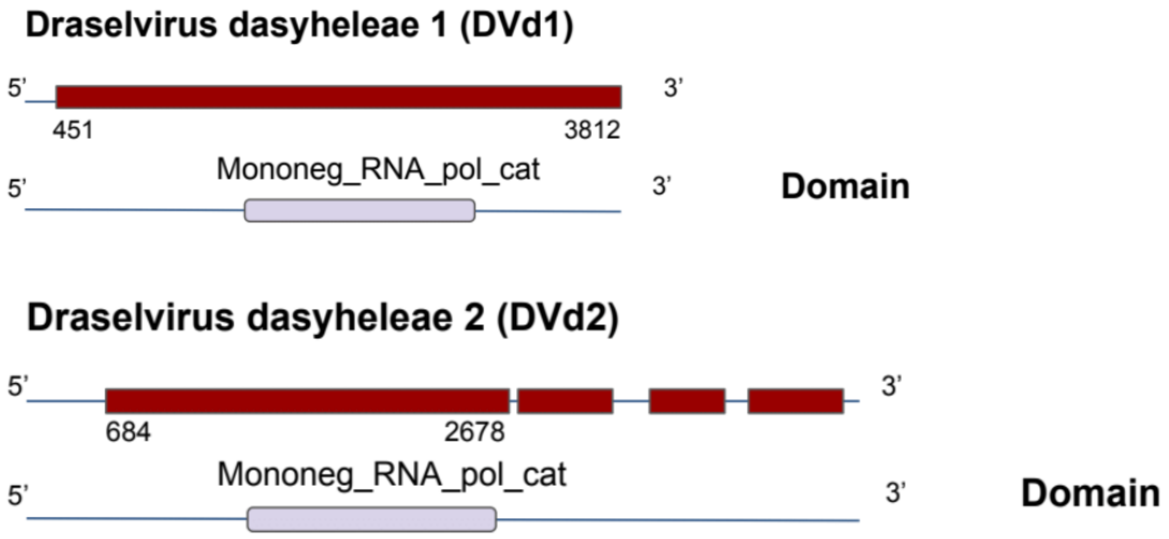

Supplementary Figure S8. Transcripts related to members of the family *Xinmoviridae*. Representation with the ORF and domains.

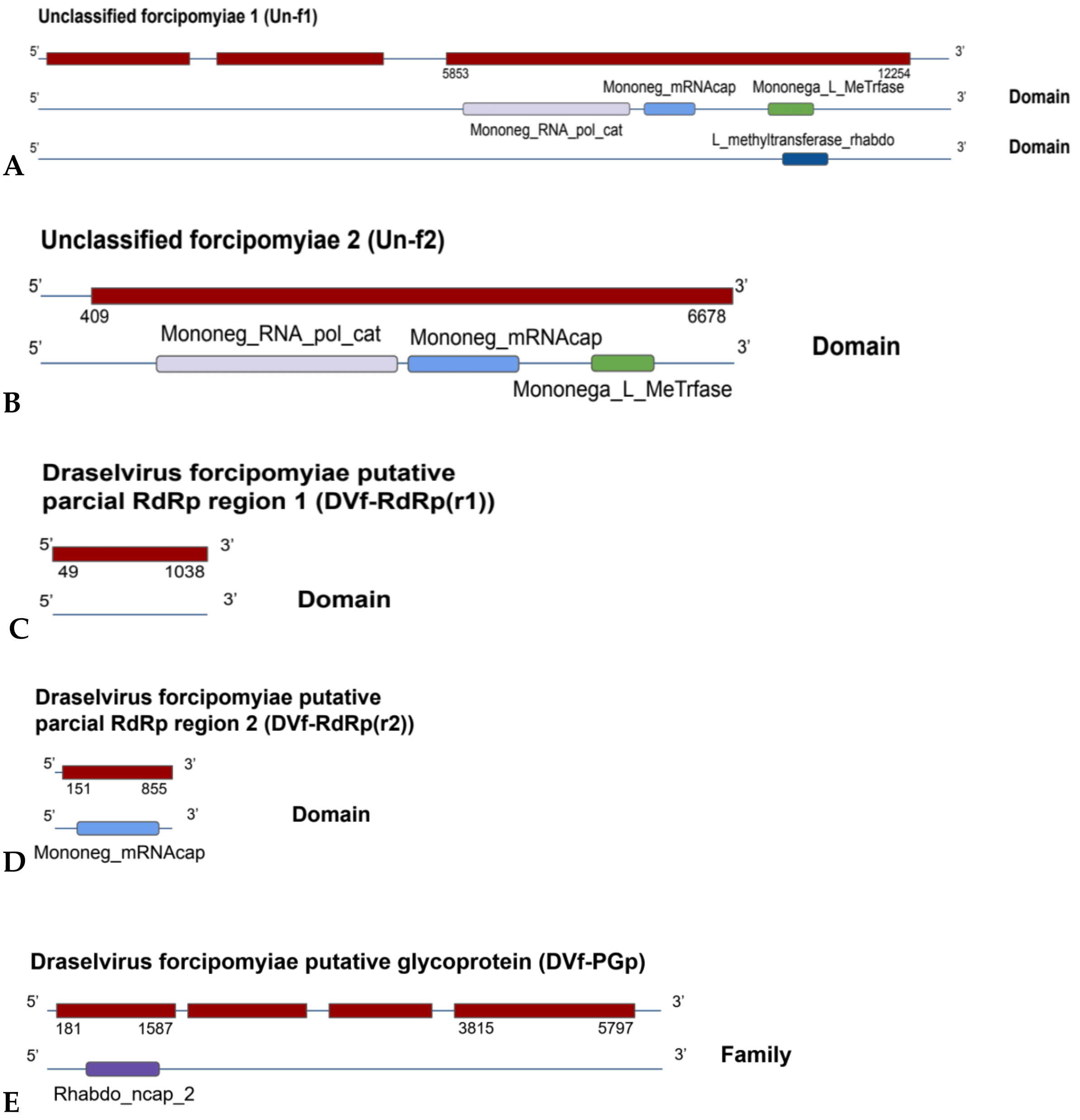

Supplementary Figure S9. Transcripts related to members of the family *Xinmoviridae*. Representation with the ORF and domains.

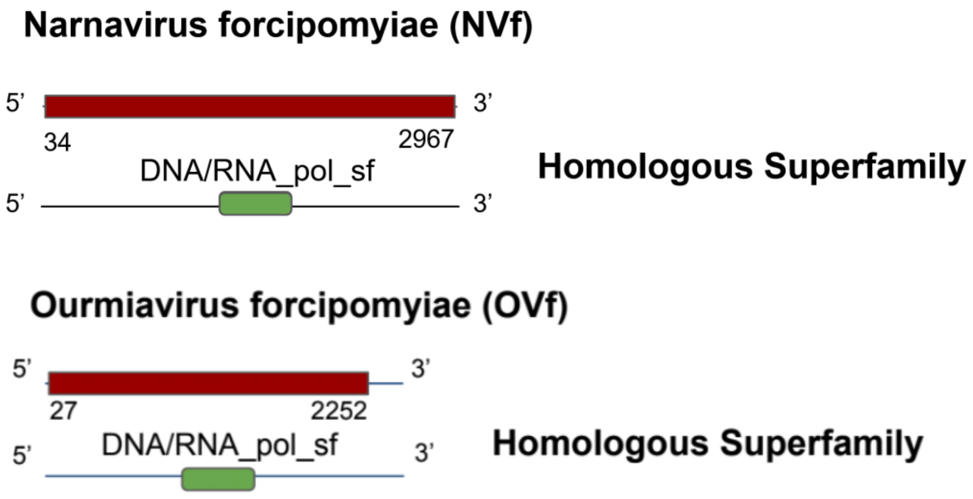

Supplementary Figure S10. Transcripts related to members of the families *Narnaviridae* and *Ourmiaviridae*. Representation with the ORF and domains.

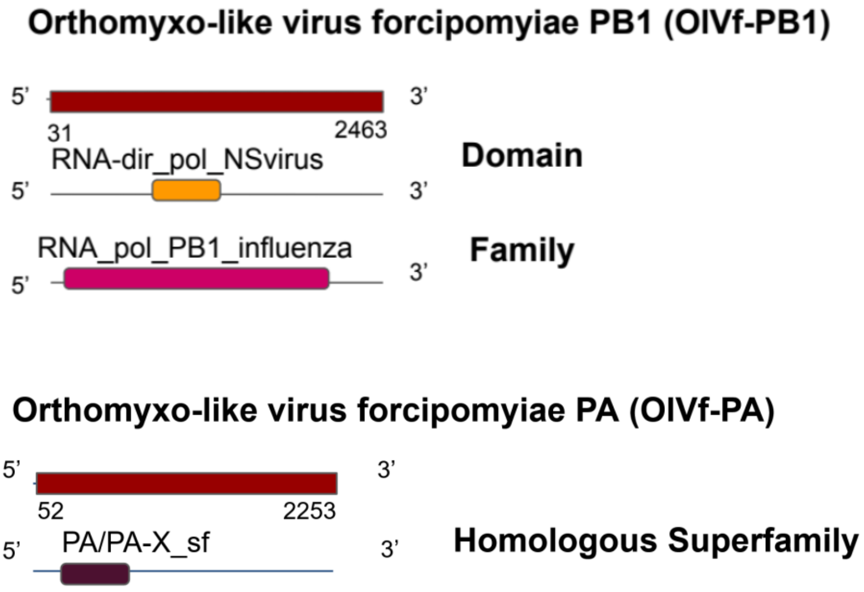

Supplementary Figure S11. Transcripts related to members of the family *Orthomyxoviridae*. Representation with the ORF and domains.
